# Hydropathy

**Published:** 1876-06

**Authors:** 


Scientific Hydropathy is no more responsible for the abuse of cold water as a remedy in disease, than are the old school doctors for the abuse of calomel by ignorant or reckless persons. In the hands of experienced men, both are remedies of very great value, and both in their places are indispensable.

Our general opinion is, that all children under ten years of age, all invalids, people of thin flesh, and those who are easily chilled, should always wash their limbs and bodies in warm water, with soap and brush, in a room almost as warm as the water itself.

